# From the Editor's Desk

**Published:** 2008-01

**Authors:** Venkataram Mysore

**Affiliations:** ***Editor-in-Chief, Journal of Cutaneous and Aesthetic Surgery***

The first issue of the *Journal of Cutaneous and Aesthetic Surgery* is in your hands, with a look that is contemporary and the contents that are relevant to today's practice of the speciality.

No other branch in dermatology has progressed as rapidly as cutaneous and aesthetic surgery in recent years. New techniques and, new technology fuelled by increasing demands of a changing society have resulted in an increasing number of dermatologists, plastic surgeons and even practitioners from other specialities taking up this speciality. Hence, the need for a journal dedicated to this branch is being felt more than ever before.

One of the criticisms, not entirely unjustified, that has been laid at the speciality of aesthetic medicine and surgery is the lack of documented evidence. It is indeed true that documentation of evidence has lagged behind introduction of new technology. This is also the situation in India and other Asian countries. There are few journals dedicated to the speciality and this journal therefore, provides a unique forum for expression and documentation of studies and other scientific matter from the region and beyond.

The newly constituted editorial board has several eminent authorities on the subject, both from India and abroad. The journal has adopted the latest practices of medical journalism such as online abstract submission, tracking and referee systems. Very soon, we hope to get it indexed also. Being one of the few open access journals in this speciality, it has the potential to be one of the top journals in the speciality. This can happen only with excellence in academic and scientific content of the journal.

I seek the cooperation of all in this exciting new endeavour.

